# Freshwater-adapted polychaetes exhibit a complete enzymatic machinery for synthesizing long-chain polyunsaturated fatty acids

**DOI:** 10.1098/rsob.250159

**Published:** 2025-09-03

**Authors:** Khalida Bainour, Nabilah Zulkifli, Ka-Kei Sam, Juan C. Navarro, Luis Filipe C. Castro, Christopher J. Glasby, Alexander C. Shu-Chien, Óscar Monroig

**Affiliations:** ^1^ Instituto de Acuicultura de Torre de la Sal (IATS), CSIC, Castellón, Spain; ^2^ Center for Chemical Biology, Universiti Sains Malaysia (USM), Bayan Lepas, Penang, Malaysia; ^3^ CIMAR/CIIMAR-Interdisciplinary Centre of Marine and Environmental Research, University of Porto, Porto, Portugal; ^4^ FCUP - Department of Biology, Faculty of Sciences, Universidade do Porto, Porto, Portugal; ^5^ Australian Museum, Sydney, Australia; ^6^ Museum and Art Gallery Northern Territory, Darwin, Australia; ^7^ Northland Aquaculture Centre, New Zealand Institute for Earth Science Ltd, Ruakaka, New Zealand

**Keywords:** Elovl, fatty acyl desaturases, freshwater polychaetes, LC-PUFA biosynthesis, *Namalycastis rhodochorde*

## Abstract

The sustainability of aquaculture is challenged by limited fishmeal and fish oil supplies, key sources of long-chain polyunsaturated fatty acids (LC-PUFA) such as eicosapentaenoic acid (EPA, 20:5 n-3), docosahexaenoic acid (DHA, 22:6 n-3) and arachidonic acid (ARA, 20:4 n-6), essential for fish health and product quality. Polychaetes represent a promising alternative. While marine polychaetes show complete LC-PUFA biosynthetic pathways involving elongases (Elovl), front-end desaturases (Fed), and methyl-end desaturases (ω des), freshwater species remain poorly studied. We hypothesize that freshwater-adapted polychaetes exhibit enhanced LC-PUFA biosynthesis to compensate for limited dietary sources in freshwater environments. This study focuses on *Namalycastis rhodochorde*, a freshwater nereid polychaete found in Southeast Asia. We isolated and characterized elongase and desaturase genes from *N. rhodochorde* using a yeast-based heterologous expression system. Our results revealed three Elovl (Elovl2/5, Elovl4, Elovl1/7) that elongate PUFA substrates from C_18_ to C_22_, two Fed (Fed1 with Δ5 and Fed2 with dual Δ6/Δ8 activities), and two ω des: a Δ12 desaturase enabling linoleic acid (18:2 n-6) synthesis, and an ω3 desaturase converting n-6 into n-3 PUFA. These findings indicate that *N. rhodochorde* has the enzymatic capacity to synthesize LC-PUFA like ARA and EPA, supporting its potential for sustainable biomass production using low-nutrient substrates.

**Figure:**
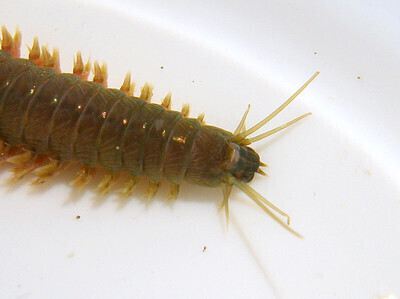

## 1. Introduction

Aquaculture is the fastest-growing food industry globally, its expansion being mostly driven by the increased production of high trophic level marine finfish species [1]. Marine fish farming relies heavily on strategically important ingredients such as fishmeal (FM) and fish oil (FO) used in feed manufacture [2]. The inclusion of FM and FO in feed formulations guarantees the provision of essential nutrients, including omega-3 (ω3 or n-3) long-chain (≥C_20_) polyunsaturated fatty acids (LC-PUFA), which are pivotal for numerous biological functions in animals [3–6]. Importantly, feeds with high contents of marine ingredients result in the high nutritional value of farmed fish products for consumers, linked to the increased levels of the health-beneficial n-3 LC-PUFA eicosapentaenoic acid (EPA, 20:5 n-3) and docosahexaenoic acid (DHA, 22:6 n-3) [7,8]. However, FM and FO are finite resources, with global annual production estimated at around 4.8 million tonnes for FM and 1 million tonnes for FO [9,10]. While a remarkable proportion of globally produced FM and FO is now derived from fisheries and aquaculture by-products [11], a significant amount of these marine ingredients is still sourced from the so-called ‘reduction fisheries’ of low trophic forage fish species [12]. Overexploitation of these fisheries has negative ecological and economic impacts [13,14]. In response, regulatory bodies have implemented strict quotas and management practices to prevent overfishing and promote sustainable utilization of reduction fisheries [15]. While these measures are mandatory for conservation, they can also limit the supply of FM and FO required to sustain the expansion of aquaculture and ultimately compromise the key role that aquaculture plays in food security [16,17]. Consequently, terrestrial-origin ingredients have been included in increasingly higher proportions in modern feed formulations, particularly in high-value species like salmonids [18–20]. However, since terrestrial raw materials are naturally poor or devoid of n-3 LC-PUFA, their use in feed formulations inevitably leads to lower nutritional value (low levels of n-3 LC-PUFA) of farmed fish products [21–23]. Alternative *de novo* sources of n-3 LC-PUFA rich products are urgently required by the aquaculture industry [24].

The abundance of EPA and DHA in animals, including fish, is mostly accounted for by the consumption of low trophic level marine organisms with the ability for LC-PUFA biosynthesis, although some fish species can biosynthesize LC-PUFA from C_18_ precursors [25]. Historically, it was believed that the primary production of n-3 LC-PUFA occurred exclusively by unicellular organisms like microalgae, protists and bacteria [26–28]. Progress to this long-lasting paradigm is the subsequent various research showing the existence of n-3 LC-PUFA biosynthesis enzymatic machinery in various marine invertebrates [29]. Biosynthesis of LC-PUFA in animals involves two key enzyme types, including elongation of very long-chain fatty acid (Elovl) proteins, responsible for extending the fatty acyl chain, and fatty acyl desaturases, responsible for the introduction of new double bonds (unsaturation). Among the latter, two sub-types can be distinguished, namely front-end desaturases (Fed) that introduce unsaturation between the carboxyl group (-COOH) and a pre-existing unsaturation, and methyl-end desaturases (ω des), which introduce new unsaturation between the methyl group (-CH_3_) and an existing one [25]. A distinctive trait of the LC-PUFA biosynthetic pathways of some invertebrate groups is the presence of ω des, which are absent in vertebrates [25]. Polychaetes are among the invertebrate phyla with characterized ω des [29,30]. Such unique LC-PUFA biosynthetic capacity, combined with the trophic flexibility of many polychaete species, which can thrive on a range of diets, from bacteria to detritus [31], opens the possibility for cultivating them on agri-food industry side-streams, under conditions that enhance their n-3 LC-PUFA biosynthesis, to obtain high-quality biomass following circular economy principles [32], this strategy represents a sustainable novel source of n-3 LC-PUFA, which is in high demand for intensive aquaculture.

Polychaetes are being explored as potential ingredients for aquafeeds due to their balanced nutritional profile, which includes high protein content and significant levels of n-3 LC-PUFA [31,33–35]. Polychaetes are commonly used as maturation diets for shrimp broodstock [36], with LC-PUFA playing pivotal roles in enhancing reproductive status in both males and females [37]. Previous studies have provided compelling evidence demonstrating that nereid polychaetes, such as the marine species *Platynereis dumerilii* and *Hediste diversicolor*, possess complete sets of enzymes enabling them to carry out *de novo* biosynthesis of LC-PUFA [29,34,38–40]. Despite their marine origin, polychaetes have demonstrated notable adaptability, successfully colonizing a range of ecological niches, including diverse freshwater habitats [41], little attention has been paid to investigating the potentially enhanced ability of freshwater species. Freshwater ecosystems are known to be relatively poor in n-3 LC-PUFA compared with marine environments [42]. Accordingly, we hypothesize that nereids adapted to freshwater habitats may have developed an increased capacity for n-3 LC-PUFA biosynthesis as an adaptive response to counterbalance the limited availability of these essential nutrients through dietary sources.

The present study aims to test this hypothesis on *Namalycastis rhodochorde*, a nereid species known for its adaptability to freshwater environments and native to the Southeast Asian shoreline [43]. *Namalycastis rhodochorde*, commonly found in sediments near Nipah trees (*Nypa fruticans*), is known as the ‘Nypa palm worm’ in Indonesia and has gained importance in the country’s emerging polychaete farming industry [44]. *N. rhodochorde* is currently harvested from the wild in its native habitat and exported alive to several countries for use as fishing bait [43], which underscores its scientific and economic importance. Here, we report the repertoire and functional characterization of elongases and desaturases that determine the LC-PUFA biosynthetic capacity of *N. rhodochorde*. Additionally, we analyse the fatty acid profile of wild-collected *N. rhodochorde* in comparison with that of *N. fruticans* and the surrounding sediment, to evaluate potential dietary sources and environmental contributions to its LC-PUFA content.

## 2. Material and methods

### 2.1. Sample collection, RNA extraction and cDNA synthesis

*Namalycastis rhodochorde* individuals were collected from their natural habitat within a Nipah mangrove in Simpang Ampat, Penang, Malaysia (coordinates: 5° 16' 15.51'' N, 100° 26' 27.47'' E). Specimens were selected for fatty acid analysis, conducted at Universiti Sains Malaysia (USM). The body was divided into four regions (head, anterior, posterior and tail) (electronic supplementary material, figure S1). The fatty acid composition was identified in each region separately and for the whole body (*n* = 3). Samples were also collected from the rachis, i.e. the main stem of the *N. fruticans* leaf, as well as from the surrounding mud at two different layers, namely, the mud top (relatively low organic matter and moisture) and the mud bottom (relatively high in organic matter), for further fatty acid analysis. The head region from another single specimen was preserved in RNAlater (20 mM EDTA, 25 mM sodium citrate, 5.3 M ammonium sulfate, pH 5.2), and subsequently shipped to the Instituto de Acuicultura Torre la Sal (IATS), Spain, for further molecular and functional characterization analyses. Following the manufacturer’s guidelines, total RNA was isolated using TRIzol reagent (Sigma-Aldrich, USA). The concentration of the extracted RNA was determined through spectrophotometric measurement (Nanodrop 2000c, ThermoFisher Scientific, USA), and an approximately 500 ng aliquot was used to analyse the RNA quality by undertaking electrophoresis on a 1% (w/v) agarose 1× TAE gel. For cDNA synthesis, 2 µg of total RNA were reverse transcribed to synthesize first-strand cDNA (Moloney murine leukaemia virus reverse transcriptase), primed with random primers and oligo-dT primers (3 : 1 mol), and following the manufacturer’s protocol (Promega, USA).

### 2.2. Retrieval of elongase and desaturase sequences from *Namalycastis rhodochorde*

Sequences of previously characterized genes encoding elongases (Elovl), Fed and ω des from the polychaete nereid *P. dumerilii* [29,39,40] were used as queries for BLAST searches (*Blastn*, E-value 1 × 10^–5^) against a transcriptome assembly of *N. rhodochorde* (A. Machado, Ó. Monroig and F. Castro, 2024, unpublished data). More specifically, the query sequences consisted of the coding regions of three Elovl (OQ731767, OQ731768, OQ731770), two Fed (OQ102603, OQ102604) and two ω des (ATV93525, ATV93530), from *P. dumerilii*. Hits that contained the full-length open reading frame (ORF) (https://www.ncbi.nlm.nih.gov/orffinder), and whose predicted protein sequences exhibited specific features of either fatty acyl elongases or desaturases [45], were retrieved for the molecular cloning of the *N. rhodochorde* elongase and desaturase genes.

### 2.3. Sequence analysis and phylogenetics of the *Namalycastis rhodochorde* elongases and desaturases

The deduced amino acid (aa) sequences of the putative *N. rhodochorde* elongases and desaturases were aligned through the MAFFT tool (https://www.ebi.ac.uk/Tools/msa/mafft/) and plotted with Color Align Conservation software (https://www.biologicscorp.com/sms2/color_align_cons.html) to highlight shared residues. The molecular features characteristic of each enzyme type were identified according to [45]. For phylogenetic analysis, three trees were constructed, each corresponding to a different type of enzyme. The deduced aa sequences from *N. rhodochorde* were compared with those from various animals (electronic supplementary material, tables S1–S3). Specifically, sequences from vertebrates such as mammals (e.g. *Homo sapiens*), teleosts (e.g. *Danio rerio*, *Salmo salar*) and amphibians (*Xenopus tropicalis*) were used. Additionally, protein sequences from invertebrates were used in the analysis, encompassing a variety of annelids including both non-clitellate annelids (i.e. the mostly marine polychaetes, e.g. *Perinereis aibuhitensis, P. dumerilii, H. diversicolor* and *Lamellibrachia satsuma*) and clitellate annelids (e.g. *Eisenia fetida*), molluscs (e.g. *Sinonovacula constricta, Octopus vulgaris, Haliotis discus hannai*), as well as crustaceans (*Echinogammarus marinus, Artemia franciscana*). A MAFFT alignment was executed using the user-customized workflow platform on the NGPhylogeny.fr server (Accessed 20/05/2024). The resulting alignment was then trimmed using trimAl to remove the columns containing gaps [46]. Subsequently, a phylogenetic tree comparing aa sequences was constructed using the maximum likelihood (ML) method [47] using MEGA X [48]. The reliability of the resulting branch topology in the phylogenetic tree was assessed through bootstrapping with 100 iterations [49].

### 2.4. Molecular cloning of the *Namalycastis rhodochorde* elongase and desaturase genes

A total of seven candidate genes, namely three Elovl (EloA, EloB, EloC), two Fed (Fed1, Fed2) and two ω des (ω des1, ω des2), were identified from *N. rhodochorde*. In order to identify their ORF sequences, polymerase chain reactions (PCR) were conducted using Prime STAR Max DNA Polymerase (Takara) and primers containing specific restriction enzyme sites to enable further cloning into the yeast expression vector pYES2 (Thermo Fisher Scientific) (table 1). The PCR programme consisted of an initial denaturation step of 30 s at 98°C, followed by 35 cycles, each consisting of 10 s at 98°C (denaturation), 15 s at the temperature specified for sequence-specific amplification (annealing) (table 1) and 20 s at 72°C (extension). The PCR procedure concluded with a final extension step of 5 min at 72°C. The PCR products were subsequently purified using the Wizard^®^ SV Gel and PCR Clean-Up System (Promega), prior digestion with the appropriate restriction enzymes (New England Biolabs) (table 1). The restricted ORF fragments were purified as above and subsequently ligated into a similarly restricted pYES2 using T4 DNA ligase (Promega). *Escherichia coli* TOP10™ competent cells (Invitrogen) were transformed with the ligation reactions, and subsequently grown overnight in Luria–Bertani (LB) agar plates with ampicillin. The colonies were screened by PCR and the positive ones were cultured overnight in LB broth containing ampicillin. Purification of the plasmids was carried out using the GenElute™ Plasmid Miniprep Kit (Sigma Aldrich, USA). The correctness of the ORF sequences was validated through Sanger DNA sequencing (DNA Sequencing Unit, IBMCP-UPV, Valencia, Spain), prior to their utilization in functional characterization assays.

**Table 1.** Primers details used in the present study. Restriction sites used for cloning into the yeast expression vector pYES2 are underlined. Ta, annealing temperature

primer name	sequence 5′ −3′	Ta (°C)	product size (bp)
Nr_Elovl2/5_VF_BamH I	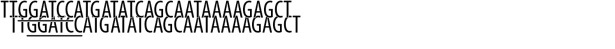	57	885
Nr_Elovl2/5_VR_XhoI	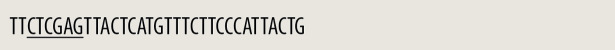		
Nr_Elovl4_VF_BamH I	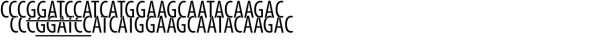	58	897
Nr_Elovl4_VR_XbaI	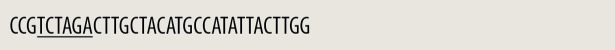		
Nr_Elovl1/7_VF_BamH I	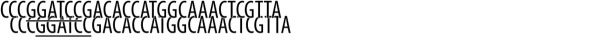	58	840
Nr_Elovl1/7_VR_XbaI	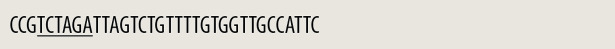		
Nr_Fed1_VF_BamH I	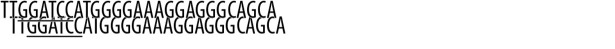	63	1326
Nr_Fed1_VR_XhoI	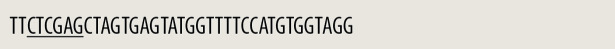		
Nr_Fed2_VF_BamH I	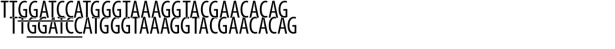	59	1317
Nr_Fed2_VR_XhoI	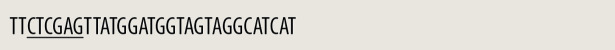		
Nr_ ω des1_VF_BamH I	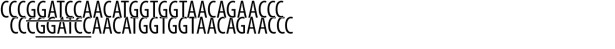	55	1158
Nr_ ω des1_VR_XbaI	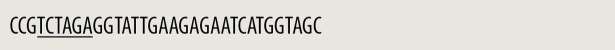		
Nr_ ω des2_VF_BamH I	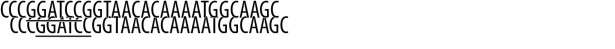	58	1257
Nr_ ω des2_VR_XbaI	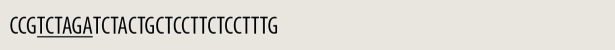		

### 2.5. Functional characterization of the *Namalycastis rhodochorde* elongases and desaturases by heterologous expression in yeast

The seven plasmid constructs (pYES2-EloA, pYES2-EloB, pYES2-EloC, pYES2-Fed1, pYES2-Fed2, pYES2-ω des1 and pYES2-ω des2) were individually transformed into competent cells of *Saccharomyces cerevisiae* (strain INV*Sc*1) using the *S.c*. EasyComp Transformation Kit (Invitrogen). The transformed yeast cells were grown on agar plates containing *S. cerevisiae* minimal medium lacking uracil (SCMM^−ura^) for 3 days at 30°C. For each enzyme assayed, one colony was grown in SCMM^−ura^ broth at 30°C for 48 h until the OD600 was between 8 and 10. An appropriate volume of the yeast bulk cultures was diluted to reach an OD600 of 0.4 in 5 ml of SCMM^−ura^ broth within a 150 ml Erlenmeyer flask. Each Erlenmeyer flask was designated for testing one potential PUFA substrate. The Erlenmeyer flasks were then incubated for 4 h at 30°C under constant shaking (250 rpm) until an OD600 of approximately 1 was reached. Subsequently, each flask was supplemented with 2% (w/v*)* galactose to induce transgene expression, as well as one of the potential PUFA substrates. The PUFA substrates used had a purity exceeding 98–99%. For the Elovl, the PUFA substrates included 18:3 n-3, 18:2 n-6, 18:4 n-3, 18:3 n-6, 20:5 n-3, 20:4 n-6, 22:5 n-3 and 22:4 n-6. For Fed, the PUFA substrates were 18:2 n-6 and 18:3 n-3 (Δ6 desaturase activity), 20:3 n-3 and 20:2 n-6 (Δ8 activity), 20:4 n-3 and 20:3 n-6 (Δ5 activity), and 22:5 n-3 and 22:4 n-6 (Δ4 activity). For ω des, the exogenously supplied PUFA substrates included 18:2 n-6 and 18:3 n-6 for Δ15 desaturase capacity, 20:2 n-6, 20:3 n-6, and 20:4 n-6 for Δ17 capacity, 22:4 n-6 and 22:5 n-6 for Δ19 capacity. To compensate for differences in the uptake efficiency of substrates with varying carbon chain lengths, PUFA substrates were added at concentrations of 0.5 mM (C_18_), 0.75 mM (C_20_) and 1.0 mM (C_22_) [34]. Controls consisting of yeast transformed with the empty pYES2 vector were also run as described above. Along with desaturation towards exogenously supplied PUFA substrates described above, the desaturase activity towards yeast endogenous fatty acids was also tested for the *N. rhodochorde* ω des1 and ω des2, as reported in previous studies (e.g. [29]). Briefly, the yeast transformed with pYES2-ω des1 or pYES2-ω des2 were grown in the absence of exogenously added fatty acid substrates and their fatty acid profiles were compared with those of control yeast (*n* = 3). Following incubation at 30°C with shaking at 250 rpm for 48 h, yeast were harvested by centrifugation for 2 min at 1500*g* washed twice with distilled water and then homogenized in a mixture of chloroform : methanol (2 : 1, v/v), with 0.01% (w/v) butylated hydroxytoluene (BHT, Sigma-Aldrich) as an antioxidant. The samples were then flushed with N_2_ and stored at −20°C for at least 24 h before fatty acid analysis.

### 2.6. Fatty acid analysis

The fatty acid analysis of yeast samples was conducted at the IATS. Total lipids were extracted from homogenized yeast samples following the Folch method [50]. The lipid extracts were used to prepare fatty acid methyl esters (FAME) through acid-catalysed transmethylation for 16 h at 50°C [51]. Subsequently, the FAME were purified using thin layer chromatography on 20 × 20 cm silica gel G60 plates (Merck KGaA, Darmstadt, Germany), and then analysed using an Agilent 6850 Gas Chromatography (GC) system coupled to a 5975 series MSD (Agilent Technologies, Santa Clara, USA). The GC was equipped with a Sapiens 5 MS (30 m × 0.25 μm × 0.25 μm) capillary column (Teknokroma), using helium as a carrier gas with a temperature ramp from 50°C to 220 °C. For elongases, the conversions of each PUFA substrate into its corresponding elongation products was calculated as the proportion of the substrate converted, using the following formula: (area of the first elongation product and longer chain products/(areas of all elongation products with longer chain than substrate+substrate area)) × 100. For desaturases, the proportion of each PUFA substrate converted into its corresponding desaturated product was determined using the formula: (desaturation product area/(desaturation product area+substrate area)) × 100. The functions of the *N. rhodochorde* ω des towards yeast endogenous fatty acids were determined by comparing the fatty acid profiles of yeast expressing each of the two ω des with those of the control yeast.

The fatty acid analysis of *N. rhodochorde* specimens and the samples collected from their surrounding environment (rachis, mud top and mud bottom) was performed at USM. Total lipids were extracted from freeze-dried samples via sonication in chloroform/methanol (2 : 1, v/v). The lipid extracts were used to prepare FAME as described previously by [52]. FAME were analysed using a GC equipped with a Supelco-2380 high-polarity fused-silica capillary column (30 m × 0.25 mm i.d. × 0.20 µm) (SGE, PA, USA) and a flame-ionization detector (GC-FID). Hydrogen was used as carrier gas with the oven thermally programmed from 80°C to 260°C at a rate of 2°C min^-1^. An additional PerkinElmer Clarus 600 GC-MS (NIST08s mass library) coupled with a PerkinElmer Elite-MS capillary column (30 m × 0.25 mm i.d. × 0.25 µm) was used to help to identify the peaks. Meanwhile, FAME were also identified by comparing the retention time to the Supelco 37 FAME standards (Sigma-Aldrich, MO, USA). The fatty acid composition of *N. rhodochorde* samples was calculated as a percentage of total fatty acids.

### 2.7. Statistics

The assays aiming to determine the ability of the *N. rhodochorde* ω des towards the yeast endogenous fatty acids were run in triplicate (*n* = 3). The homogeneity of variances was checked using Levene’s test. The fatty acid profiles from control yeast and those from yeast expressing the ω des from *N. rhodochorde* were compared using the Student’s *t*‐test. A one-way analysis of variance (ANOVA), followed by Tukey’s honest significant difference (HSD) test, was conducted to assess differences in fatty acid composition across different body regions. Statistical analyses, including principal component analysis (PCA), were employed to analyse and visualize the relationship between the body region and the fatty acid profiles of *N. rhodochorde*. All statistical analyses were carried out using SPSS software (version 15.0 for Windows; IBM, USA). Statistical significance was set at *p* < 0.05.

## 3. Results

### 3.1. Phylogeny and sequence analysis of the *Namalycastis rhodochorde* elongases

**Figure 1.**
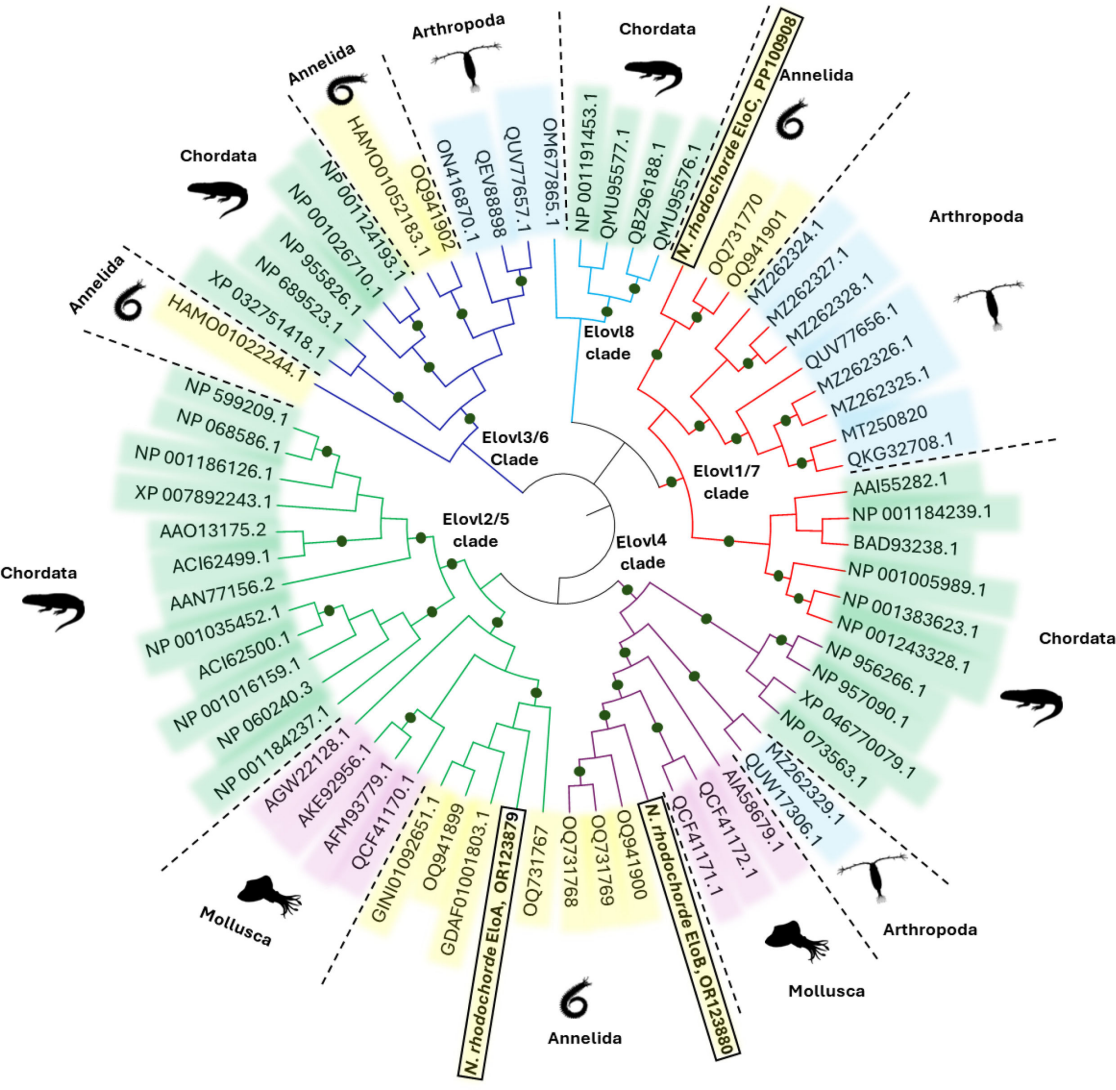
Phylogenetic tree comparing the three elongases (EloA, EloB and EloC) retrieved from *N. rhodochorde*, with Elovl-like sequences from other animals including vertebrates and invertebrates. The tree was constructed using the maximum likelihood (ML) method [47] using MEGA-X software [48]. Coloured dots on the nodes represent supporting values of 80% or above for ML. For each sequence, the accession numbers are given according to the information contained in the corresponding NCBI databases. These results show that EloA is an Elovl2/5, EloB is an Elovl4 and EloC is an Elovl1/7. Further details associated with the accession numbers shown in the tree can be found in electronic supplementary material, table S1.

To validate the orthology of each of the identified *N. rhodochorde* elongase sequences, we generated ML phylogenetic trees, with various sequence representatives of vertebrates, annelids, molluscs and crustaceans (figure 1). The results revealed that the *N. rhodochorde* EloA group together with Elovl2/5 from other nereid polychaetes, as well as molluscs, and, more distantly, to Elovl2 and Elovl5 sequences from vertebrates (figure 1). The *N. rhodochorde* EloB grouped with Elovl4 sequences from vertebrates and invertebrates including polychaetes, crustaceans and molluscs (figure 1). Regarding the *N. rhodochorde* EloC, the putative protein clustered with Elovl1/7 sequences from polychaetes, crustaceans, as well as Elovl1 and Elovl7 from vertebrates (figure 1). Our analysis shows that the isolated elongases are bona fide orthologues, with EloA being an Elovl2/5, EloB an Elovl4 and EloC an Elovl1/7. The sequence analyses demonstrated that the putative aa sequences deduced from the *N. rhodochorde* Elovl genes have characteristic features of microsomal fatty acyl elongases, including highly conserved motifs such as KXXEXXDT, QXXFLHXXHH (which contains the histidine box ‘HXXHH’), NXXXHXXMYXYY and TXXQXXQ (electronic supplementary material, figure S2). The ORF sequences of the *N. rhodochorde* Elovl2/5, Elovl4 and Elovl1/7 consist of 885, 897 and 840 bp, respectively, encoding putative proteins of 295, 299 and 280 aa (electronic supplementary material, figure S2). These sequences have been deposited in the GenBank database with accession numbers OR123879 (Elovl2/5), OR123880 (Elovl4) and PP100908 (Elovl1/7).

### 3.2. Phylogeny and sequence analysis of the *Namalycastis rhodochorde* front-end desaturases

**Figure 2.**
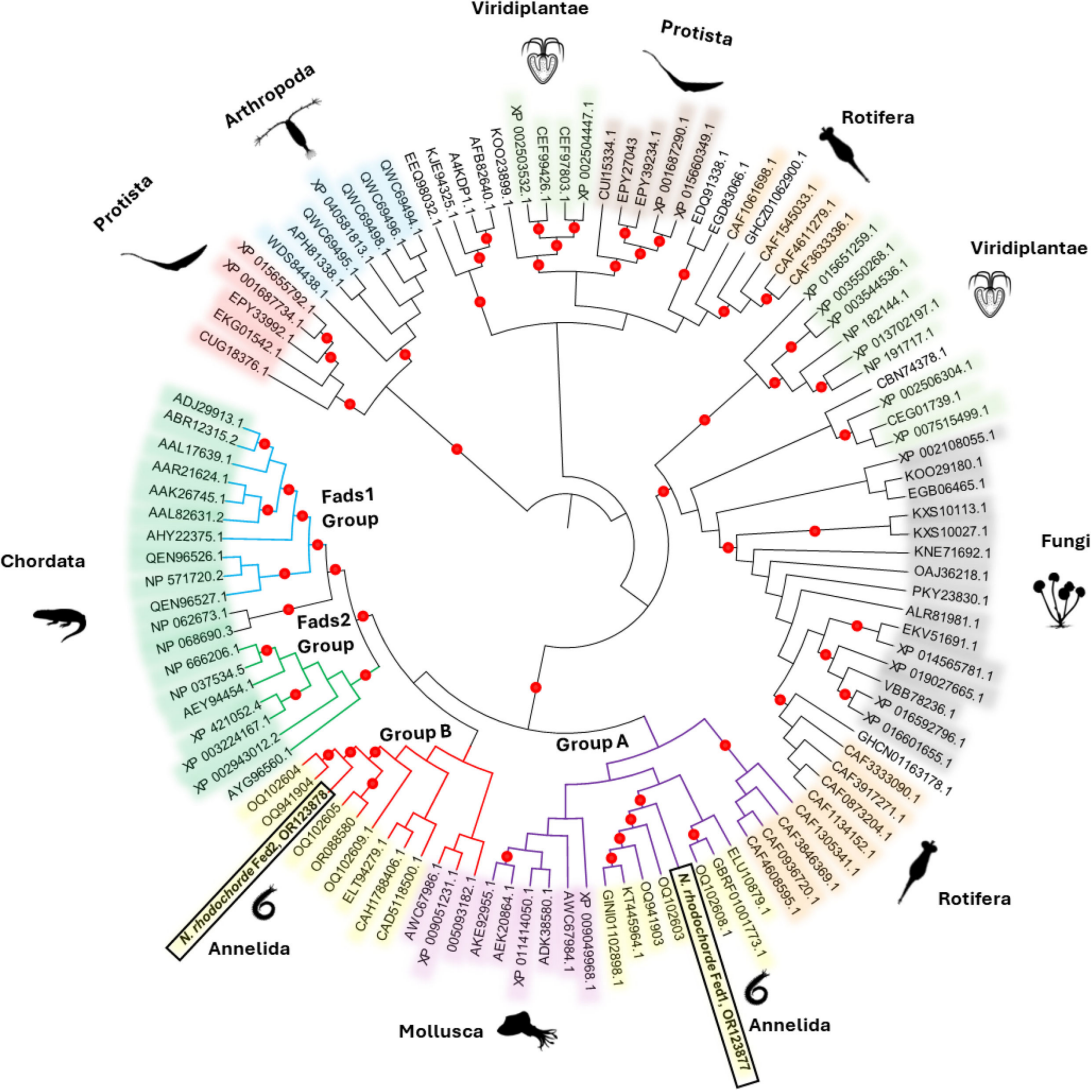
Phylogenetic tree comparing the two front-end desaturases (Fed1 and Fed2) retrieved from the *N. rhodochorde* transcriptome, with Fed-like sequences from other organisms. The tree was constructed using the maximum likelihood method [47] using MEGA-X software [48]. Coloured dots on the nodes represent supporting values of 80% or above for ML. For each sequence, the accession numbers are given according to the information contained in the corresponding NCBI databases. Further details associated with the accession numbers shown in the tree can be found in electronic supplementary material, table S2.

The two Fed from *N. rhodochorde*, herein termed as Fed1 and Fed2, were categorized into two primary groups, referred to as Group A and Group B (figure 2), following the nomenclature established by [53,54] for molluscs and cnidarians. Group A included the *N. rhodochorde* Fed1 with other sequences from annelids, molluscs and rotifers, some being reported as Δ5 desaturases (figure 2). Moreover, Group B included the *N. rhodochorde* Fed2 and desaturase sequences with Δ6 and Δ8 activities from other invertebrates such molluscs and annelids (figure 2). Our sequence analysis showed that the deduced aa sequences of Fed1 and Fed2 contain all the conserved motifs characteristic of front-end desaturases, including a cytochrome b5-domain with the haem-binding motif (HPGG) and three H-box (HXXXH, HXXHH and QXXHH) (electronic supplementary material, figure S3). Notably, the third H-box begins with a glutamine (Q) residue, consistent with motifs defined for front-end desaturases by [45]. The ORF sequences of the *N. rhodochorde* Fed1 and Fed2 contain 1326 and 1317 bp, respectively, encoding proteins of 442 and 439 aa (electronic supplementary material, figure S3). These sequences have been deposited in the GenBank database with accession numbers OR123877 for Fed1 and OR123878 for Fed2.

### 3.3. Phylogeny and sequence analysis of the *Namalycastis rhodochorde* methyl-end desaturases

**Figure 3.**
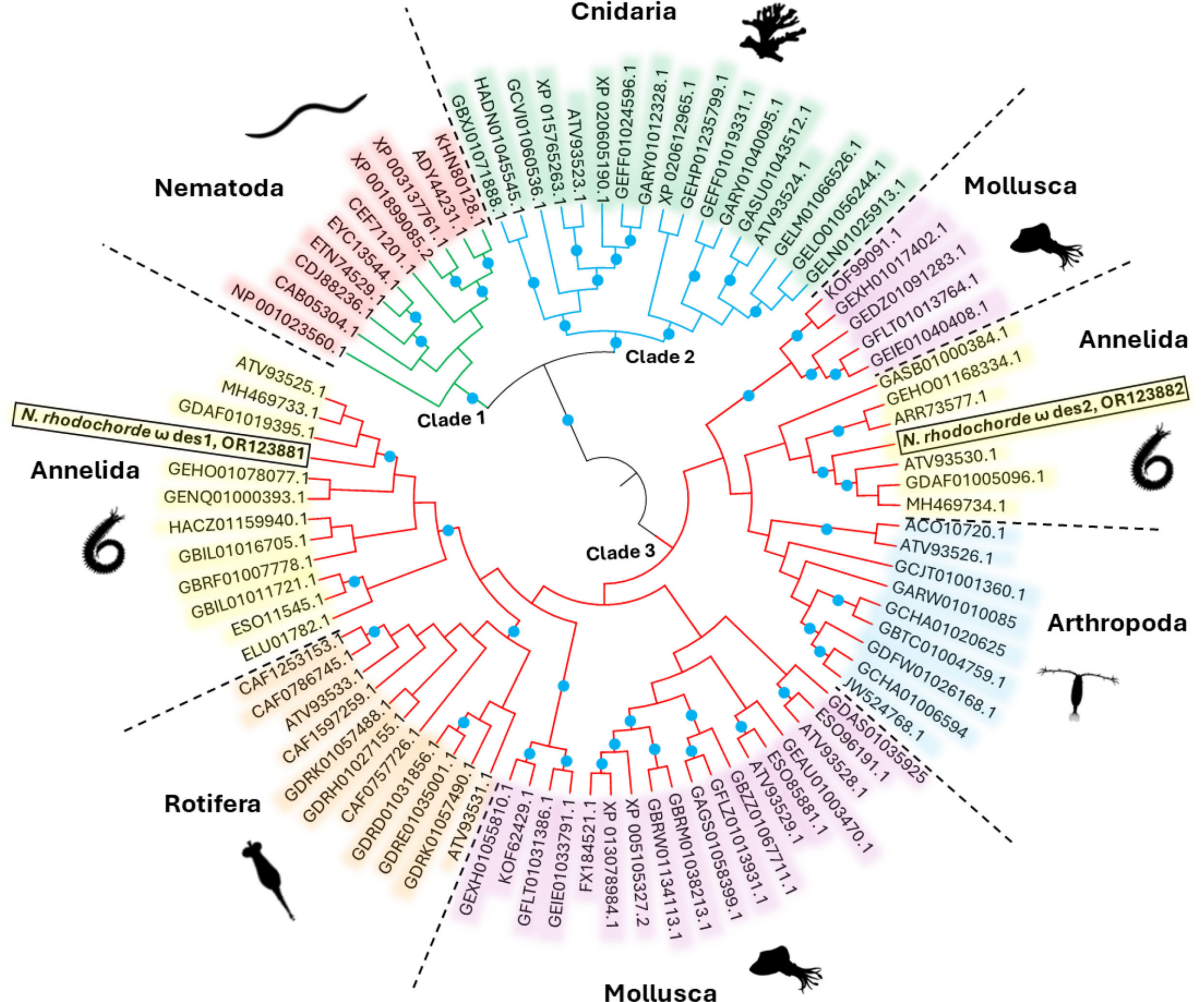
Phylogenetic tree comparing the two methyl-end desaturases (ω des1 and ω des2) retrieved from *N. rhodochorde*, with ω des-like sequences from other animals including vertebrates and invertebrates. The tree was constructed using the maximum likelihood method [47] using MEGA-X software [48]. Coloured dots on the nodes represent supporting values of 80% or above for ML. For each sequence, the accession numbers are given according to the information contained in the corresponding NCBI databases. Further details associated with the accession numbers shown in the tree can be found in electronic supplementary material, table S3.

Our phylogenetic analysis showed that both ω des1 and ω des2 group within Clade 3, encompassing sequences from a variety of invertebrates including rotifers, molluscs and arthropods, along with other annelids (figure 3). Other well-defined clusters included Clade 1, comprising ω des sequences from nematodes, and Clade 2, which includes ω des sequences from cnidarians (figure 3). Within Clade 3, sequences from Annelida are divided into two moderately supported clades: one clade includes *N. rhodochorde* ω des1, along with the functionally characterized Δ12 desaturase from *P. dumerilii* (93525.1), while the other clade comprises *N. rhodochorde* ω des2 (MH469734.1), along with the functionally characterized ω3 desaturases from *H. diversicolor* (figure 3). The deduced aa sequences of the *N. rhodochorde* ω des1 and ω des2 feature three H-box (HXXXH, HXXHH and HXXHH) (electronic supplementary material, figure S4), and lack a cytochrome b5 domain. The ORF sequences of the *N. rhodochorde* ω des1 and ω des2 consist of 1158 and 1257 bp, respectively, encoding putative proteins of 386 and 419 aa (electronic supplementary material, figure S4). These sequences have been deposited in GenBank under accession numbers OR123881 (ω des1) and OR123882 (ω des2).

### 3.4. Functions of the *Namalycastis rhodochorde* elongases in long-chain polyunsaturated fatty acids biosynthesis

The functionality of the *N. rhodochorde* Elovl2/5, Elovl4 and Elovl1/7 enzymes was characterized by analysing the fatty acid profiles of *S. cerevisiae* expressing their ORF and grown in the presence of putative elongation substrates. The results demonstrated that the *N. rhodochorde* Elovl2/5 has relatively high affinity (45.1–75.2%) towards C_18_ and C_20_ PUFA substrates (table 2). This enzyme displayed relatively lower activity (≤7.3%) towards C_22_ substrates, namely 22:5 n-3 and 22:4 n-6 (table 2). The *N. rhodochorde* Elovl2/5 showed elongation capacity towards monounsaturated fatty acids (MUFA), as observed by the detection of elongation products of both n-7 and n-9 series originated from the yeast endogenous 16:1 n-7 and 18:1 n-9, respectively (data not shown). Moreover, both Elovl4 and Elovl1/7 from *N. rhodochorde* were able to elongate all C_18_, C_20_ and C_22_ PUFA substrates (table 2). Importantly, yeast expressing the *N. rhodochorde* Elovl4 were able to produce 26:5 n-3 and 26:4 n-6 when grown in the presence of 22:5 n-3 and 22:4 n-6, respectively (table 2).

**Table 2.** Functional characterization of the *N. rhodochorde* elongases Elovl2/5 (herein termed ‘EloA’), Elovl4 (‘EloB’) and Elovl1/7 (‘EloC’). Conversions of polyunsaturated fatty acids used as substrates were calculated according to the formula (area of the first elongation product and longer chain products/(areas of all elongation products with longer chain than substrate+substrate area)) × 100. nd, Not detected

substrate	product	conversion (%)			activity
		Elovl2/5	Elovl4	Elovl1/7	
18:3 n-3	20:3 n-3	64.8	22.9	7.2	C_18_ → C_20_
	22:3 n-3	6.5	8.2	21.8	C_20_ → C_22_
18:2 n-6	20:2 n-6	66.9	1.1	2.9	C_18_ → C_20_
	22:2 n-6	13.3	nd	8.1	C_20_ → C_22_
18:4 n-3	20:4 n-3	75.2	11.1	20.8	C_18_ → C_20_
	22:4 n-3	13.2	8.7	6.3	C_20_ → C_22_
18:3 n-6	20:3 n-6	65.9	4.2	10.5	C_18_ → C_20_
	22.3 n-6	18.3	6.2	1.9	C_20_ → C_22_
20:5 n-3	22.5 n-3	45.1	12.7	29.3	C_20_ → C_22_
	24:5 n-3	3.2	4.8	38.0	C_22_ → C_24_
20:4 n-6	22:4 n-6	68.2	6.0	16.6	C_20_ → C_22_
	24:4 n-6	4.8	4.1	8.9	C_22_ → C_24_
22:5 n-3	24:5 n-3	3.3	3.3	15.8	C_22_ → C_24_
	26:5 n-3	nd	3.4	nd	C_24_ → C_26_
22:4 n-6	24:4 n-6	7.3	2.4	4.2	C_22_ → C_24_
	26:4 n-6	nd	8.5	nd	C_24_ → C_26_

### 3.5. Functions of the *Namalycastis rhodochorde* front-end desaturases in long-chain polyunsaturated fatty acid biosynthesis

The functional characterization of the *N. rhodochorde* Fed1 and Fed2 was carried out by determining the conversions that yeast expressing their ORF were able to carry out towards Δ6 (18:3 n-3 and 18:2 n-6), Δ5 (20:3 n-3 and 20:4 n-3), Δ4 (22:5 n-3 and 22:4 n-6) and Δ8 desaturation (20:3 n-3 and 20:2 n-6) substrates. For Fed1, only the exogenously added substrates 20:4 n-3 and 20:3 n-6 were converted into the desaturated products 20:5 n-3 and 20:4 n-6, respectively. These results indicate that the *N. rhodochorde* Fed1 has Δ5 desaturation activity (table 3). No evidence of activity as Δ4, Δ6 or Δ8 desaturase was observed for the *N. rhodochorde* Fed1 (table 3). With regard to Fed2, transgenic yeast expressing its ORF efficiently desaturated the substrates 18:3 n-3 and 18:2 n-6 to 18:4 n-3 and 18:3 n-6, respectively, confirming that the encoded protein has Δ6 desaturase activity (table 3). Furthermore, the *N. rhodochorde* Fed2-transformed yeast demonstrated the ability to desaturate 20:3 n-3 and 20:2 n-6 to 20:4 n-3 and 20:3 n-6, respectively, indicating that *N. rhodochorde* Fed2 also has Δ8 activity (table 3). These results show that *N. rhodochorde* Fed2 is a dual Δ6/Δ8 desaturase. No Δ4 or Δ5 desaturase activities were detected for the *N. rhodochorde* Fed2 (table 3).

**Table 3.** Functional characterization of the *N. rhodochorde* front-end desaturases Fed1 and Fed2 in yeast. Conversions of polyunsaturated fatty acids used as substrates were calculated according to the formula (desaturation product area/(desaturation product area+substrate area)) × 100. The Δ desaturase activity assayed for each substrate is indicated accordingly. nd, Not detected

substrate	product	conversion (%)		activity
		Fed1	Fed2	
18:3 n-3	18:4 n-3	nd	1.1	Δ6
18:2 n-6	18:3 n-6	nd	1.4	Δ6
20:3 n-3	20:4 n-3	nd	50.5	Δ8
20:2 n-6	20:3 n-6	nd	54.0	Δ8
20:4 n-3	20:5 n-3	0.4	nd	Δ5
20:3 n-6	20:4 n-6	0.1	nd	Δ5
22:5 n-3	22:6 n-3	nd	nd	Δ4
22:4 n-6	22:5 n-6	nd	nd	Δ4

### 3.6. Functions of the *Namalycastis rhodochorde* methyl-end desaturases in long-chain polyunsaturated fatty acid biosynthesis

**Figure 4.**
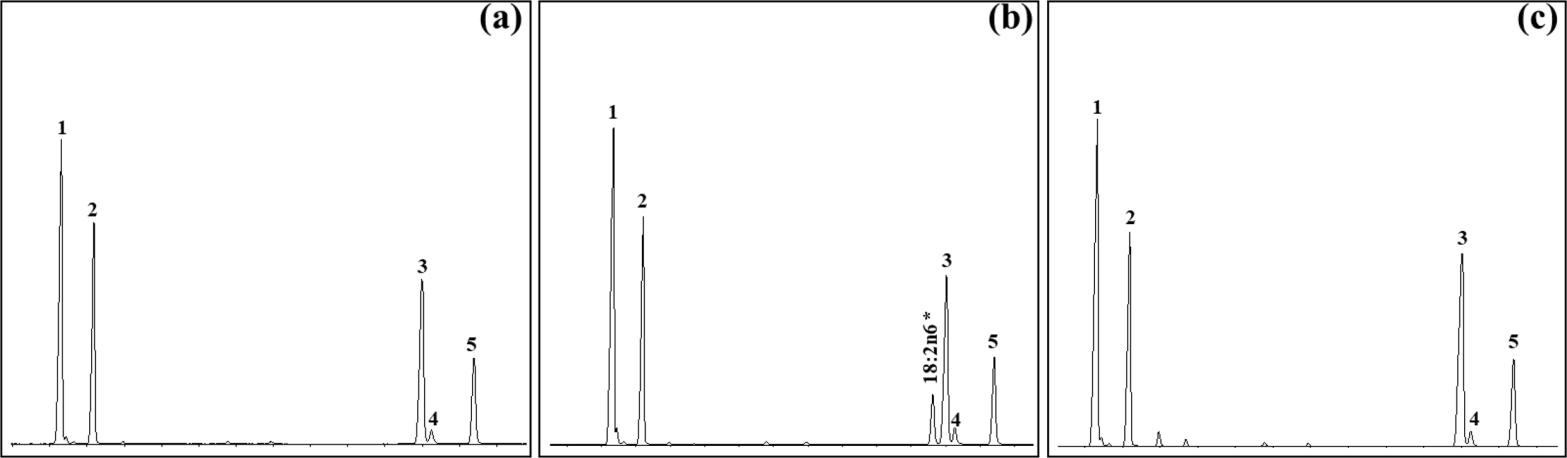
Representative chromatograms of FAME samples prepared from the transgenic yeast transformed, in the absence of exogenously supplied substrates, with (a) control (empty pYES2), (b) the *N. rhodochorde* ω des1 and (c) the *N. rhodochorde* ω des2. The yeast endogenous fatty acids including 16:1 n-7, 16:0, 18:1 n-9, 18:1 n-7, and 18:0 are indicated as 1−5 in all panels.

First, the desaturase activity of the newly cloned *N. rhodochorde* ω des1 and ω des2 was assessed by comparing the fatty acid profiles of yeast transformed with pYES2-ω des1 and pYES2-ω des2 with those of control yeast (transformed with empty pYES2). The fatty acid profiles of the control yeast exhibited prominent peaks corresponding to 16:0, 16:1 n-7, 18:0 and 18:1 isomers (18:1 n-9 and 18:1 n-7), which are consistent with the lipid composition of wild-type *S. cerevisiae* (figure 4). While the ω des2 from *N. rhodochorde* did not display activity towards yeast endogenous fatty acids (figure 4c), yeast expressing the *N. rhodochorde* ω des1 contained a prominent peak identified as 18:2 n-6, in addition to yeast endogenous fatty acids (figure 4b). These results clearly show that the *N. rhodochorde* ω des1 has Δ12 desaturase activity. We further assayed the desaturase activities of the *N. rhodochorde* ω des1 and ω des2 towards a range of n-6 substrates including 18:2 n-6, 18:3 n-6, 20:2 n-6, 20:3 n-6, 20:4 n-6, 22:4 n-6 and 22:5 n-6 (table 4). The *N. rhodochorde* ω des2 demonstrated the ability to synthesize n-3 PUFA from their corresponding n-6 precursors of C_18_, C_20_ and C_22_, revealing that this enzyme is an ω3 desaturase with Δ15, Δ17 and Δ19 activities, respectively (table 4). By contrast, the *N. rhodochorde* ω des1 showed no detectable activity towards any of the n-6 substrates assayed (table 4).

**Table 4.** Functional characterization of the *N. rhodochorde* methyl-end desaturases ω des1 and ω des2. Conversions of polyunsaturated fatty acid*s* used as substrates were calculated according to the formula (desaturation product area/(desaturation product area+substrate area)) × 100. The Δ desaturase activity assayed for each substrate is indicated accordingly. nd, Not detected.

substrate	product	conversion (%)		activity
		ω des1	ω des2	
18:2 n-6	18:3 n-3	nd	5.6	Δ15
18:3 n-6	18:4 n-3	nd	2.3	Δ15
20:2 n-6	20:3 n-3	nd	22.6	Δ17
20:3 n-6	20:4 n-3	nd	36.2	Δ17
20:4 n-6	20:5 n-3	nd	41.8	Δ17
22:4 n-6	22:5 n-3	nd	23.6	Δ19
22:5 n-6	22:6 n-3	nd	nd	Δ19

### 3.7. Fatty acid profiles of wild-captured *Namalycastis rhodochorde* and surrounding environment

**Figure 5.**
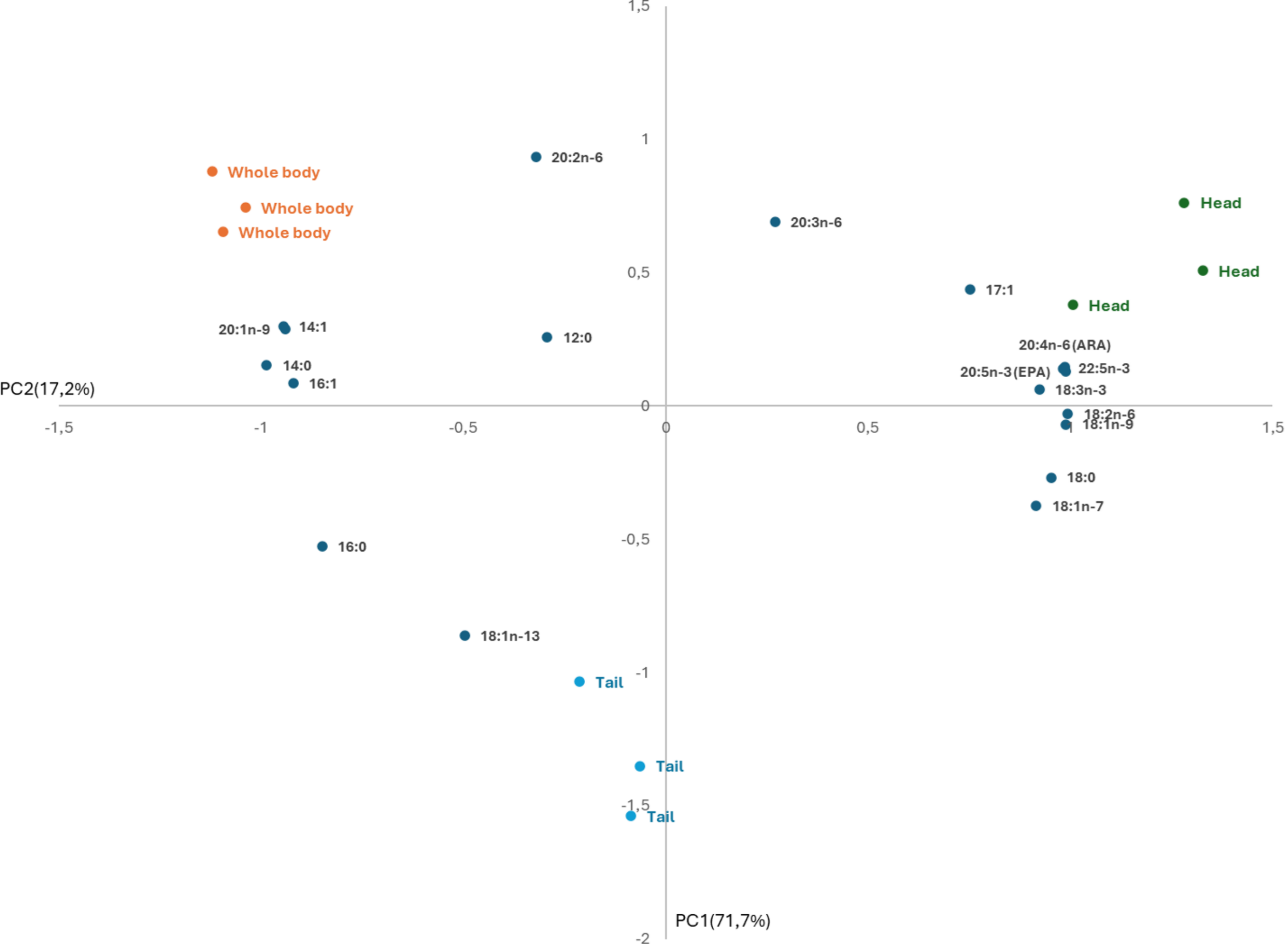
PCA of the fatty acid composition of *N. rhodochorde* across different body regions (head, tail and whole body). Green, orange and blue circles represent the fatty acid profiles of the head, whole body and tail regions, respectively.

The fatty acid profile of *N. rhodochorde* shows a clear dominance of saturated fatty acids (SFA), particularly 14:0 and 16:0, across all body regions (electronic supplementary material, table S4). MUFA make up around 19–21%, with notable contributions from 16:1 and 18:1 n-9 (electronic supplementary material, table S4). PUFA are more prominent in the head region (27.3%) due to the presence of ARA (20:4 n-6) and EPA (20:5 n-3) (electronic supplementary material, table S4). Notably, DHA (22:6 n-3) was not detected in any body region (electronic supplementary material, table S4). For the PCA, only the head, tail and whole-body regions were selected, as the anterior and posterior regions showed fatty acid profiles similar to the whole-body samples (electronic supplementary material, table S4). The PCA revealed that the first principal component (PC1) accounted for 71.7% of the variance, while the second component (PC2) explained 17.2% of the variance in the dataset (figure 5). The PCA loading plot shows distinct patterns of fatty acid distribution across these body regions of *N. rhodochorde*. SFA, such as 16:0 and 14:0, and MUFA, like 14:1, 16:1 and 20:1 n-9, are positioned on the negative side of PC1, strongly associated with the whole-body samples (figure 5). By contrast, the tail region shows a strong association with 18:1 n-13 on the negative side of PC2 (figure 5). On the positive side of PC1, PUFA, such as ARA and EPA, are grouped particularly aligning with the head region (figure 5).

The fatty acid profiles of rachis and mud (top and bottom) representing the nutritional environment where *N. rhodochorde* inhabits, are included in electronic supplementary material, table S5. SFA are the most prominent fatty acid group in all samples, with 16:0 being a major component. MUFA are present, with notable levels of 18:1 n-9 (electronic supplementary material, table S5). While short-chain (C_18_) PUFA are also detected, the presence of ARA and EPA was not detected in rachis or mud (electronic supplementary material, table S5).

## 4. Discussion

In light of the recognized importance of LC-PUFA for optimal fish growth, there is an increasing need to explore alternatives to traditional FM due to economic factors and limited availability. Polychaetes have garnered attention in this respect owing to their high content of LC-PUFA [35,55]. While the LC-PUFA biosynthesis has been extensively explored in marine polychaetes [29,34,38–40], little attention has been paid to freshwater species despite their potentially higher LC-PUFA biosynthetic capacity associated with decreased dietary input in freshwater ecosystems. As a first step to explore this hypothesis, we examined the complement and functions of elongases and desaturases operating the LC-PUFA biosynthetic pathways of the freshwater-adapted polychaete *N. rhodochorde*. Our findings demonstrate that *N. rhodochorde* has three elongases, two front-end desaturases, and two methyl-end desaturases enabling biosynthesis of LC-PUFA such as ARA and EPA.

The phylogenetic analysis of the *N. rhodochorde* elongases clarified their categorization as Elovl2/5, Elovl4 and Elovl1/7. This PUFA elongase pattern is consistent with that reported in marine polychaetes [34,52]. However, crustaceans have both Elovl4 and Elovl1/7, but notably lack Elovl2/5 [25]. Molluscs, on the other hand, possess Elovl2/5 and Elovl4, though the presence of Elovl1/7 remains to be confirmed [53]. The putative Elovl2/5, Elovl4 and Elovl1/7 from *N. rhodochorde* exhibit characteristic motifs common to elongases, including conserved domains KXXEXXDT, NXXXHXXMYXYY, QXXFLHXXHH (which contains the histidine box ‘HXXHH’) and TXXQXXQ [56]. Relevant to LC-PUFA biosynthesis, the three *N. rhodochorde* Elovl contain a glutamine (Q) in position −5 from the H-box, a trait that distinguishes PUFA elongases from non-PUFA elongases [45]. These findings strongly suggest that the *N. rhodochorde* Elovl2/5, Elovl4 and Elovl1/7 are PUFA elongases, and thus play relevant roles in LC-PUFA biosynthesis. This was confirmed in the yeast-based functional assays.

The functional analysis of the *N. rhodochorde* Elovl2/5 demonstrated its elongation activity towards C_18_ and C_20_ PUFA substrates as shown previously for marine polychaetes [34,39]. A distinctive trait of the *N. rhodochorde* Elovl2/5 from marine nereids [34,39] is its affinity towards C_22_ PUFA substrates that were bioconverted to C_24_ PUFA products. Among the characterized Elovl2/5 enzymes, only the European amphioxus *Branchiostoma lanceolatum*, but not molluscs, has shown the ability to elongate C_22_ PUFA substrates [25]. Along Elovl2/5, the *N. rhodochorde* Elovl4 and Elovl1/7 elongases are also involved in the LC-PUFA biosynthesis as demonstrated by their elongation affinity towards C_18_, C_20_ and C_22_ PUFA substrates. Again, these results are in agreement with those reported in marine annelids [34,39]. It is important to note that, in addition to its role in LC-PUFA biosynthesis, the *N. rhodochorde* Elovl4 is further involved in the synthesis of the so-called ‘very long-chain (>C_24_) polyunsaturated fatty acids’. Indeed, the functional assays carried out in the present study revealed that the *N. rhodochorde* Elovl4 can produce 26:5 n-3 and 26:4 n-6 by stepwise elongation of 22:5 n-3 and 22:4 n-6, respectively. While similar roles of Elovl4 have been reported in crustaceans [25], such an elongation capability by the *N. rhodochorde* Elovl4 has not yet been reported in marine polychaetes [34,39], thus posing the question of whether this Elovl4 capability is unique from freshwater species.

In coordinated action with elongases, fatty acyl desaturases play pivotal roles in LC-PUFA biosynthesis in animals. Here, we provide compelling evidence that *N. rhodochorde* has two front-end desaturases (Fed1 and Fed2) with well-defined functions in the LC-PUFA biosynthesis. First, the *N. rhodochorde* Fed1 belongs to the Group A cluster, which includes functionally characterized Δ5 front-end desaturases found in various animals such as molluscs [25]. Consistently, annelids, including nereid polychaetes, possess one sole Group A-like desaturase with Δ5 desaturase activity, as reported here for the *N. rhodochorde* homologue [34,40]. The functional analysis of *N. rhodochorde* Fed1 has shown that this enzyme enables the biosynthesis of ARA and EPA from 20:3 n-6 and 20:4 n-3, respectively. By contrast to functions reported for other polychaete Δ5 desaturases, the *N. rhodochorde* could not biosynthesize non-methylene interrupted fatty acids (NMI-FA) as reported in other annelids [34,40]. The apparent inability of *N. rhodochorde* Fed1 to biosynthesize NMI-FA may be attributed to the particularly low conversions observed in the yeast assay, which could hinder the detection of NMI-FA produced by the Δ5 desaturase activity. NMI-FA are commonly found in the lipids of various marine invertebrates such as sponges, molluscs, echinoderms and annelids [57]. However, they appear to be absent in certain freshwater clitellates like *Lumbriculus variegatus* and *Tubifex tubifex* [58], as well as in the terrestrial clitellate *E. fetida* [59–61]. A further front-end desaturase found in *N. rhodochorde* (herein termed ‘Fed2’) clustered in Group B containing functionally characterized Δ6/Δ8 desaturases from a variety of polychaete annelids like *H. diversicolor* [34] and clitellate such as *Hirudo medicinalis* and *E. fetida* [40]. In agreement, the *N. rhodochorde* Fed2 demonstrated dual Δ6 and Δ8 activities since it was able to convert, respectively, 18:2 n-6 and 18:3 n-3 to 18:3 n-6 and 18:4 n-3 (Δ6 desaturations), and 20:2 n-6 and 20:3 n-3 to 20:3 n-6 and 20:4 n-3, respectively (Δ8 desaturations). These activities are consistent with those found in the phylogenetically unrelated front-end desaturase reported in the copepod *Tigriopus californicus* Δ6/Δ8, suggesting a case of convergent evolution [25].

The present study demonstrated the presence of two functionally active ω des enzymes (ω des1 and ω des2) in *N. rhodochorde*. The phylogenetic analysis showed that both *N. rhodochorde* ω des clustered within the previously termed Clade 3 [29], which encompasses sequences primarily from Lophotrochozoa (molluscs, rotifers and annelids). The support resolution of the tree within Clade 3 does not allow us to fully clarify the phylogenetic relationship of the two herein-characterized *N. rhodochorde* ω des enzymes. More clearly though, the occurrence of two distinct ω des-encoding genes arises as a well conserved pattern among nereid polychaetes according to investigation on *P. dumerilii* [29] and *H. diversicolor* [38]. Possessing two ω des genes is a remarkable physiological advantage, particularly where the encoded enzymes exhibit distinct but complementary functions, as demonstrated for *N. rhodochorde*. Certainly, our functional experiments have unequivocally shown the ability of *N. rhodochorde* for *de novo* synthesis of PUFA and, from them, LC-PUFA. Specifically, the *N. rhodochorde* ω des1 is a Δ12 desaturase catalysing the conversion of the MUFA 18:1 n-9 (oleic acid, OA) into the PUFA 18:2 n-6 (linoleic acid, LA). Similar findings were observed in *P. dumerilii* [29], although the *H. diversicolor* ω des1 enzyme was able to recognize a more varied range of fatty acid substrates [38]. Regarding the *N. rhodochorde* ω des2, this enzyme has an ω3 desaturase regioselectivity as it was able to desaturate multiple n-6 PUFA (18:2 n-6, 18:3 n-6, 20:2 n-6, 20:3 n-6, 20:4 n-6 and 22:4 n-6) into their corresponding ω3 PUFA products. These conversions showcase the Δ15, Δ17 and Δ19 desaturation capabilities of the *N. rhodochorde* ω des2, which were also found, among other invertebrates, in homologous enzymes from *P. dumerilii* [29] and *H. diversicolor* [38]. However, the *N. rhodochorde* ω des2 could not convert 22:5 n-6 into 22:6 n-3 (DHA), which contrasts with the ability of the *H. diversicolor* ω3 desaturase [38]. The above results highlight a remarkable diversity in substrate specificity of ω des within the nereid family.

**Figure 6.**
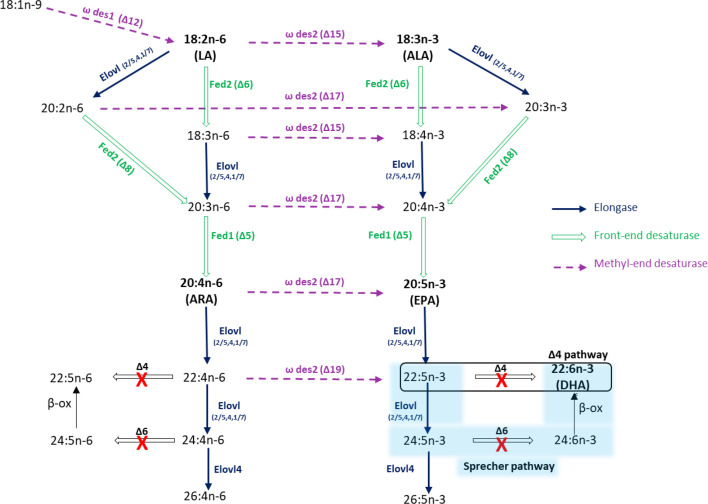
Biosynthetic pathways of LC-PUFA in *N. rhodochorde*. Elongation steps mediated by the herein characterized elongases are labelled as ‘Elovl’ and indicated in blue arrows. Reactions catalysed by methyl-end desaturases identified in *N. rhodochorde* are shown in purple dashed arrows, while those catalysed by front-end desaturases are marked in green. The red ‘X’ indicates enzyme activities that are absent in this polychaete.

The combined evidence from the analysis of elongases and desaturases in *N. rhodochorde* indicates that this polychaete can biosynthesize LC-PUFA, including ARA and EPA. The dataset from functional characterization allowed us to predict the LC-PUFA biosynthetic pathways, illustrated in figure 6. The biosynthesis pathways begin with a Δ12 desaturation (ω des1), converting OA (18:1 n-9) into LA (18:2 n-6). LA can undergo further desaturation by Δ15 desaturase (ω des2), resulting in α-linolenic acid (ALA, 18:3 n-3). The pathway starts with the Δ6 (Fed2) desaturation of LA or ALA, followed by elongation and Δ5 (Fed1) desaturation, leading to the production of ARA and EPA, respectively (figure 6). Alternative pathways may involve elongating LA or ALA to eicosadienoic acid (20:2 n-6) or eicosatrienoic acid (20:3 n-3), which then undergo Δ8 (Fed2) and Δ5 desaturations to yield ARA and EPA (figure 6). Our findings demonstrate that *N. rhodochorde* lacks the Δ4 desaturase activity required for converting 22:5 n-3 into DHA via the ‘Δ4 pathway’, as observed in certain teleosts and copepods [25]. However, *N. rhodochorde* possesses elongases (Elovl2/5, Elovl4, Elovl1/7) that potentially support DHA biosynthesis through the Sprecher pathway by producing 24:5 n-3 via direct elongation of 22:5 n-3 or stepwise elongation of EPA. Crucially, this pathway requires a Δ6 desaturase to convert 24:5 n-3 into 24:6 n-3, which is then transformed into DHA via β-oxidation [62]. While this mechanism is functional in vertebrates [62–64], the inability of Δ6 desaturase in *N. rhodochorde* to desaturate C_24_ fatty acids (data not shown) suggests a limitation in the full utilization of the Sprecher pathway for DHA synthesis (figure 6). Similarly, other annelids also lack both Δ4 desaturases for direct DHA conversion and functional Δ6 desaturases for the Sprecher pathway [40]. Nonetheless, fatty acid profile analysis of *N. rhodochorde* individuals collected from the wild revealed significant levels of LC-PUFA, including ARA and EPA. Remarkably, ARA and EPA are absent from *N. fruticans* and surrounding sediment where it is commonly found (electronic supplementary material, table S5). These findings strongly support the conclusion that *N. rhodochorde* can biosynthesize LC-PUFA through the elongases and desaturases, as demonstrated in this study and highlight the polychaete’s ability to produce LC-PUFA independently of dietary sources, an important adaptation for survival in nutrient-limited environments. The absence of DHA in *N. rhodochorde* aligns with studies on marine polychaetes that report moderate levels of DHA typically found in wild-collected specimens [65,66] and confirms the lack of both Δ4 and Δ6 desaturase activities. Although *N. rhodochorde* shows limited DHA synthesis, its endogenous production of EPA and ARA, and the presence of methyl-end desaturases, highlight its potential ecological and biotechnological role in freshwater aquaculture. These polychaetes may help convert n-6 rich plant-based feed residues into n-3 LC-PUFA, potentially contributing to trophic upgrading in pond ecosystems [67]. Furthermore, promoting European freshwater fish ponds as living laboratories could help uncover similar LC-PUFA biosynthetic capacities in other benthic taxa, including invasive polychaetes like *Hypania invalida* and common but understudied macrozoobenthic organisms such as *Procambarus virginalis, Limnodrilus hoffmeisteri* and *Chironomus plumosus* [68].

In conclusion, this study successfully identified and characterized three Elovl (Elovl2/5, Elovl4 and Elovl1/7), two Fed (Δ5 and Δ6/Δ8) and two ω des (Δ12 and ω3) from the nereid annelid *N. rhodochorde*. Functional analysis revealed that Elovl2/5, Elovl4 and Elovl1/7 in *N. rhodochorde* act as PUFA elongases, demonstrating an affinity towards C_18_, C_20_ and C_22_ PUFA substrates. The Δ5 desaturase activity of *N. rhodochorde* Fed1 facilitates the biosynthesis of ARA and EPA from 20:3 n-6 and 20:4 n-3, respectively. Fed2, on the other hand, exhibits dual Δ6 and Δ8 desaturase activities, thus complementing those of Fed1. *N. rhodochorde* possesses two ω des, with one acting as a monofunctional Δ12 desaturase and the other as an ω3 desaturase capable of desaturating C_18_, C_20_ and C_22_ n-6 PUFA (figure 6). Overall, the *in vitro* functional assays demonstrate that *N. rhodochorde* possesses complete LC-PUFA biosynthetic pathways enabling this polychaete to convert dietary short-chain, poorly unsaturated fatty acids into high nutritional value LC-PUFA, including ARA and EPA. However, future research under *in vivo* conditions is necessary to evaluate the enzymatic activity of desaturases and elongases in *N. rhodochorde*, and to compare their expression levels and substrate specificities with those of marine polychaetes. Such comparisons could help determine whether this freshwater species possesses enhanced or divergent LC-PUFA biosynthetic capacities. Furthermore, studies investigating the regulation of these metabolic pathways under different environmental or dietary conditions, similar to those conducted in marine polychaetes [69], could be useful to understand how LC-PUFA biosynthesis is modulated *in vivo*, particularly in nutrient-poor habitats where dietary sources of these essential fatty acids are limited.

## Data Availability

All data produced in the present study have been presented in the submitted manuscript and associated supplementary materials [70].
